# Supplementary material for: Collapse and resurgence of the Iceland mantle plume
Source: Nat Commun. 2026 May 2;17:4104. doi: 10.1038/s41467-026-71618-2 (PMC13149877; doi:10.1038/s41467-026-71618-2)
Supplement: Supplementary file 1 — Supplementary Information [file 41467_2026_71618_MOESM1_ESM.pdf]

Supplementary Information for Pearman et al. (2026): Collapse and resurgence of the Iceland mantle plume

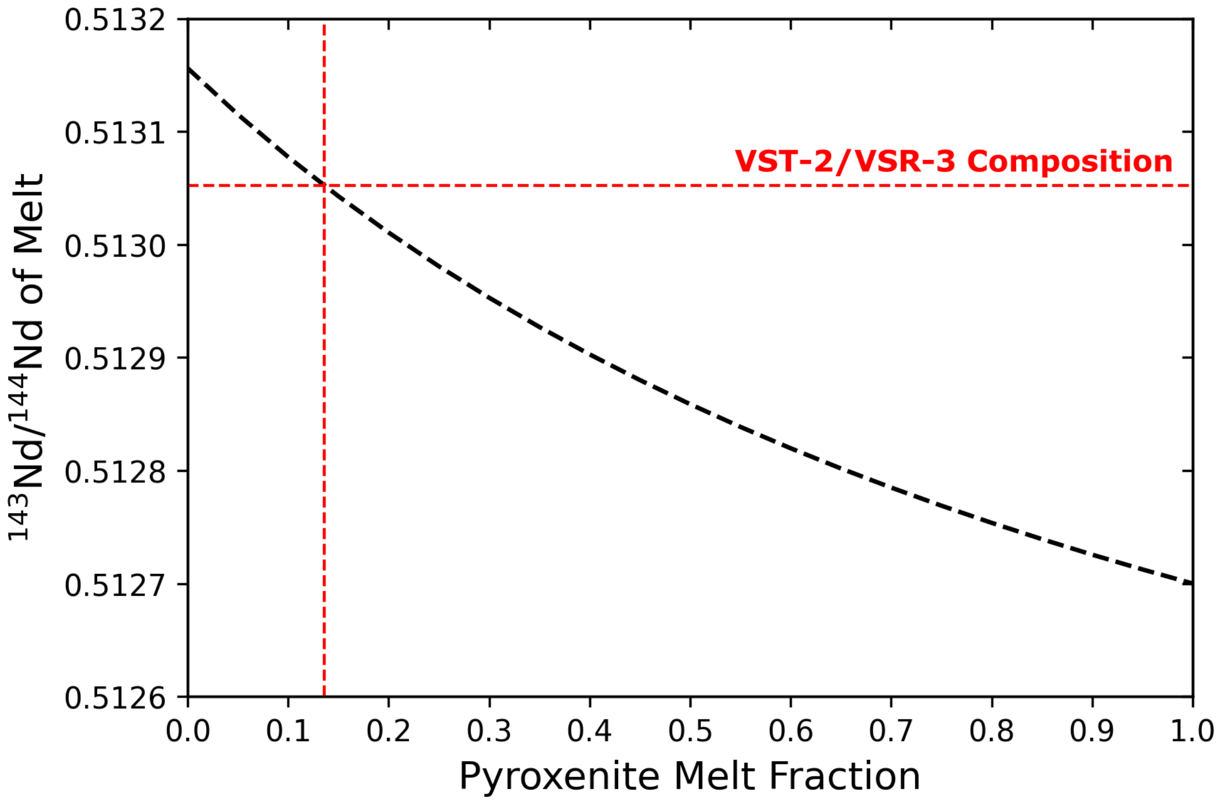

Supplementary Figure 1. Isotopic mixing relationship. ^{143}Nd/^{144}Nd of melt plotted as function of pyroxenite melt fraction used to calculate effect of adding isotopically enriched pyroxenite melt to spinel lherzolite melt. Isotopic compositions of lherzolite and pyroxenite components estimated from axial dredges where flowline intersects mid-ocean ridge (Figures 1b and 3) and from Koornneef et al. (2012) [1], respectively. Black dashed line is calculated using standard binary relationship where Nd concentration of lherzolite mantle source component is that of depleted mantle component of plume, which is similar to DMM of Workman & Hart (2005) [2], and Nd concentration of pyroxenite mantle source component is given by 1:1 ratio of DMM and recycled oceanic crust [3]. Pair of red dashed lines show inferred pyroxenite melt fraction for ^{143}Nd/^{144}Nd compositions of VST-2 and VSR-3. Nd concentration of melts calculated using VSR-3 pyMelt model (1420°C, 2.6% Px).

References

1. Koornneef, J. M. *et al.* Melting of a two-component source beneath Iceland. *J. Petrol.* **53,** 127–157 (2012).
2. Workman, R. K. & Hart, S. R. Major and trace element composition of the depleted MORB mantle (DMM). *Earth Planet. Sci. Lett.* **231,** 53–72 (2005).
3. Stracke, A., Bizimis, M. & Salters, V. J. M. Recycling oceanic crust: Quantitative constraints. *Geochem. Geophys. Geosyst.* **4** (2003).

List of IODP Expedition 395 Science Party: Ship- and Shore-based

Name	Affiliation
Anne Briais	Geo-Ocean, Centre National de la Recherche Scientifique (CNRS), Institut Universitaire Européen de la Mer, Rue Dumont d'Urville, Plouzané, France
Ying Cui	Department of Earth and Environmental Studies, Montclair State University, 1 Normal Ave., Montclair, NJ, USA
Anita Di Chiara	Istituto Nazionale di Geofisica e Vulcanologia, Via di Vigna Murata 605, Roma, Italy
Justin P. Dodd	Texas A&M University, Department of Oceanography, 3146 TAMU, O&M 315, College Station, TX 77843-3146, USA
Deepa Dwyer	College of Earth, Ocean and Atmospheric Sciences, Oregon State University, 1500 SW Jefferson Way, Corvallis, OR, USA
Sarah A. Friedman	School of Earth, Environment & Sustainability, Georgia Southern University, Statesboro, GA, USA
Sidney R. Hemming	Department of Earth and Environmental Sciences, Columbia University, New York, NY, USA; Lamont-Doherty Earth Observatory, Columbia University, Palisades, NY, USA
Katharina Hochmuth	Institute of Marine and Antarctic Studies (IMAS), College of Science and Engineering, University of Tasmania, Hobart, Australia
Halima E. Ibrahim	Department of Earth Sciences, Binghamton University, Binghamton, NY, USA
Claire E. Jasper	Department of Earth and Environmental Sciences, Columbia University, New York, NY, USA; Lamont-Doherty Earth Observatory, Columbia University, Palisades, NY, USA; now at Ocean Sciences Department, University of California, Santa Cruz, Santa Cruz, CA, USA
Tom Dunkley Jones	School of Geography, Earth and Environmental Sciences, University of Birmingham, Birmingham, United Kingdom
Boris-Theofanis Karatsolis	Department of Earth Sciences, Uppsala University, Villavägen 16, 752 36, Uppsala, Sweden; now at Department of Geosciences, University of Fribourg, Fribourg, Switzerland
Saran Lee-Takeda	Atmosphere and Ocean Research Institute, The University of Tokyo, Kashiwa, Chiba, Japan
Danielle E. LeBlanc	Woods Hole Oceanographic Institution, MA, USA
Leah J. LeVay	International Ocean Discovery Program, Texas A&M University, 1000 Discovery Drive, College Station, TX, USA
Melody R. Lindsay	Bigelow Laboratory for Ocean Sciences, 60 Bigelow Drive, East Boothbay, ME, USA
David D. McNamara	Department of Earth, Ocean and Ecological Sciences, University of Liverpool, 4 Brownlow Street, Liverpool, United Kingdom
Sevasti E. Modestou	Department of Geography and Environmental Sciences, Northumbria University, Newcastle upon Tyne, United Kingdom
Margaret A. Morris	Institute of Geophysics & Planetary Physics, Scripps Institution of Oceanography, University of California, San Diego, La Jolla, CA, USA
Bramley J. Murton	National Oceanography Centre, European Way, Southampton, United Kingdom
Suzanne O'Connell	Department of Earth and Environmental Sciences, Wesleyan University, 265 Church Street, Middletown, CT, USA
Ross Parnell-Turner	Institute of Geophysics & Planetary Physics, Scripps Institution of Oceanography, University of California, San Diego, La Jolla, CA, USA
Gabriel Pasquet	Complex Fluids and Reservoirs Laboratory, University of Pau and Pays de l'Adour, Avenue de l'Université, Pau, France; now at Bureau of Economic Geology, Jackson School of Geosciences, The University of Texas at Austin, USA
Paul N. Pearson	Department of Earth Sciences, University College London, Gower Street, London, United Kingdom
Sheng-Ping Qian	Southern Marine Science and Engineering Guangdong Laboratory (Guangzhou), Guangzhou, China
Yair Rosenthal	Department of Marine and Coastal Sciences, Rutgers, The State University of New Jersey, 71 Dudley Road, New Brunswick, NJ, USA
Sara Satolli	Department of Engineering and Geology, University of Chieti-Pescara, Via dei Vestini 31, Chieti, Italy
Matthias Sinnesael	Geology, School of Natural Sciences, Trinity College Dublin, The University of Dublin, College Green, Dublin, Ireland
Takuma Suzuki	SUGAR, X-star, Japan Agency for Marine-Earth Science and Technology (JAMSTEC), Yokosuka, Japan
Thena Thulasi	Geosciences Division, National Centre for Polar and Ocean Research (NCPOR), Vasco-da-Gama, Goa, India
Nicky White	Bullard Laboratories, Department of Earth Sciences, University of Cambridge, Madingley Road, Cambridge, United Kingdom
Tao Wu	Ocean College, Zhejiang University, Zhoushan, China
Alexandra Y. Yang	Guangzhou Institute of Geochemistry, Chinese Academy of Sciences, 511 Kehua Street, Tianhe District, Guangzhou, Guangdong, China
Callum Pearman	Bullard Laboratories, Department of Earth Sciences, University of Cambridge, Madingley Road, Cambridge, United Kingdom
Chia-Yu Tien	Bullard Laboratories, Department of Earth Sciences, University of Cambridge, Madingley Road, Cambridge, United Kingdom
Viviane dos Santos Rocha	Earth, Atmosphere and Environment, Northern Illinois University, 180 Stadium Drive, DeKalb, IL, USA
